# Treatment and prevention of epilepsy in onchocerciasis-endemic areas is urgently needed

**DOI:** 10.1186/s40249-024-01174-8

**Published:** 2024-01-12

**Authors:** Robert Colebunders, Joseph Nelson Siewe Fodjo, Olivia Kamoen, Luís-Jorge Amaral, Amber Hadermann, Chiara Trevisan, Mark J. Taylor, Julia Gauglitz, Achim Hoerauf, Yasuaki Sato, Katja Polman, María-Gloria Basáñez, Dan Bhwana, Thomson Lakwo, Gasim Abd-Elfarag, Sébastien D. Pion

**Affiliations:** 1 Global Health Institute, University of Antwerp, Antwerp, Belgium; 2 Department of Neurology, Heilig Hart Ziekenhuis, Lier, Belgium; 3 Department of Public Health, Institute of Tropical Medicine, Antwerp, Belgium; 4 Liverpool School of Tropical Medicine, Liverpool, UK; 5 Department of Computer Science, University of Antwerp, Antwerp, Belgium; 6 Institute of Medical Microbiology, Immunology and Parasitology, University Hospital Bonn, Bonn, Germany; 7 German Center for Infection Research (DZIF), Partner Site Bonn-Cologne, Bonn, Germany; 8 School of Global Humanities and Social Sciences, Nagasaki University, Nagasaki, Japan; 9 MRC Centre for Global Infectious Disease Analysis and London Centre for Neglected Tropical Disease Research, Department of Infectious Disease Epidemiology, School of Public Health, Imperial College London, London, UK; 10 National Institute for Medical Research, Tanga, Tanzania; 11 Vector Control Division, Ministry of Health, Kampala, Uganda; 12 School of Public Health, University of Juba, Juba, South Sudan; 13 Access for Humanity, Juba, South Sudan; 14 French National Research Institute for Sustainable Development, Montpellier, France

**Keywords:** Onchocerciasis, Morbidity, Burden of disease, Prevention, Ivermectin, Epilepsy, Nodding syndrome, Treatment, Children, Policy

## Abstract

**Background:**

There is increasing epidemiological evidence supporting the association between onchocerciasis and seizures, reinforcing the concept of onchocerciasis-associated epilepsy (OAE). The aim of this paper is to provide an update on the new knowledge about OAE and to propose recommendations to the World Health Organization how to address this public health problem.

**Main text:**

During the 2nd International Workshop on OAE held on 19–21 September, 2023, in Antwerp, Belgium, participants recognised OAE as a substantial yet neglected public health problem, particularly in areas of sub-Saharan Africa where onchocerciasis remains hyperendemic. Evidence from prospective population-based studies suggest that strengthening onchocerciasis elimination efforts leads to a significant reduction of OAE incidence. There is a need to validate an OAE case definition to estimate the burden of disease and identify onchocerciasis-endemic areas requiring intensification of onchocerciasis elimination programmes and integration of epilepsy care. It is expected that raising awareness about OAE will boost the population uptake of ivermectin. The implementation of a community-based epilepsy treatment programme offering free anti-seizure medications (ASMs) has shown high effectiveness in reducing the frequency of seizures and improving the overall quality of life of people with epilepsy.

**Conclusions:**

To reduce OAE burden, enhanced collaboration between onchocerciasis and mental health programmes at community, national, and international levels is required. Urgent efforts are needed to ensure the uninterrupted provision of free ASMs in onchocerciasis-endemic areas. Furthermore, OAE should be included in the quantification of the onchocerciasis disease burden.

**Graphical Abstract:**

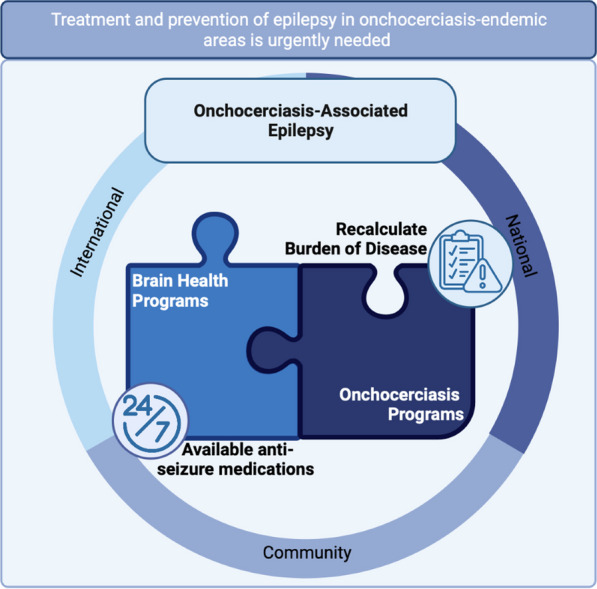

## Background

Over the past decades, numerous studies have consistently reported a higher epilepsy prevalence in areas with high onchocerciasis transmission [1]. Growing epidemiological evidence suggests that, in such settings, onchocerciasis may directly or indirectly induce seizures, leading to the concept of onchocerciasis-associated epilepsy (OAE) [2]. Individuals with OAE present with a broad spectrum of seizures, including head-nodding seizures (nodding syndrome) [2]. OAE is frequently linked to cognitive decline, behavioural and psychiatric issues, developmental delays, disabilities, severe stigma and premature mortality. Current estimates indicate that more than 300,000 people suffer from OAE [3].

In October 2017, an inaugural international workshop on OAE was held in Antwerp (Belgium) to establish a scientific network to draw attention to this neglected public health problem. Since then, much progress has been made in OAE epidemiology and prevention. However, there are still outstanding research questions.

The 2nd International Workshop on OAE was held in Antwerp on 19–21 September 2023. This (hybrid) workshop brought together 113 participants from 24 countries, including 14 African countries. The workshop aimed at reviewing recent findings from OAE studies and proposing recommendations for the World Health Organization (WHO).

### The burden of disease caused by onchocerciasis

The disease burden attributed to onchocerciasis, currently assessed by mild-to-severe skin disease, visual impairment and blindness, is likely underestimated by not including OAE. Comprehensive reviews of population-based studies show that, in areas of high *Onchocerca volvulus* transmission, the OAE prevalence [1] can be as high as the prevalence of onchocerciasis-related blindness [4].

### Need for a validated OAE case definition

A practical OAE case definition has been used to identify OAE hotspots and estimate its burden [5]. A person with epilepsy is suspected to present OAE if the following criteria were met: (1) epilepsy onset between that ages of three and 18 years, (2) no obvious cause of epilepsy (e.g. perinatal asphyxia, history of severe malaria, measles, encephalitis or meningitis, or head injury), (3) living for at least three years in a meso- or hyperendemic onchocerciasis area with geographic and familial clustering of epilepsy cases with similar characteristics, and (4) normal neurological development before the onset of epilepsy. Additional criteria suggesting OAE include: a history of head nodding seizures, cognitive impairment, stunted growth and laboratory or clinical evidence of an *O. volvulus* infection.” However even with these additional criteria it is always possible the epilepsy may be relate to another infective cause. Due to its limited specificity, this definition cannot be relied upon for epidemiological, clinical and therapeutic purposes. The specificity of the OAE definition depends on the level of onchocerciasis endemicity and the prevalence of other epilepsy aetiologies in the area. An essential criterion in the case definition is the exclusion of other apparent causes of epilepsy through history-taking and clinical examination. In onchocerciasis-endemic areas, where neuroimaging and electroencephalogram capabilities are generally unavailable, misclassification of underlying aetiologies of epilepsy is very likely. Neurocysticercosis, a primary cause of epilepsy, may hamper the use of the OAE definition in *Taenia solium*–*O. volvulus* co-endemic areas. Nevertheless, the current OAE definition may prove highly valuable in settings such as South Sudan, where there are nearly no pigs and neurocysticercosis is unlikely.

In pathogenesis studies, it is advisable to use a more specific clinical-based presentation of OAE, such as nodding syndrome. Pending a biomarker or test to confirm OAE diagnosis, it becomes crucial to develop policies to prevent and treat OAE where its clinical manifestations are found. For instance, an increased supply of anti-seizure medications (ASMs) should be provided to hospitals and healthcare facilities located in onchocerciasis-endemic areas. These areas often experience a high epilepsy prevalence due to OAE, leading to high demand of ASMs.

### Onchocerciasis-associated epilepsy can be prevented

Although the pathogenesis of OAE is still under investigation, two retrospective cohort studies in Cameroon have shown a dose-response relationship between *O. volvulus* microfilarial intensity in childhood and the risk of developing epilepsy later in life [6]. Other retrospective studies in Uganda have suggested that mass drug administration with ivermectin may prevent OAE [7]. Two prospective population-based studies, one conducted in Maridi, South Sudan, and the other in Mahenge, Tanzania, reported that strengthening onchocerciasis elimination efforts, including introducing biannual (six-monthly) community-directed treatment with ivermectin (CDTI), resulted in significant reductions in OAE and nodding syndrome incidence [8]. Therefore, ensuring high ivermectin coverage, particularly in the 5–15 years age group appears crucial to prevent the development of OAE.

In situations where six-monthly CDTI is not feasible, an extra round of ivermectin distribution in schools could be considered. This school-based approach could be integrated with existing child deworming programmes. Additionally, to increase treatment coverage among women of childbearing age, ivermectin could be given to women at child vaccination sites, at least one week after delivery, if they missed a CDTI round due to pregnancy. Such an approach, combined with an OAE awareness programme, has recently proven effective in Maridi, South Sudan (S. Jada, personal communication). Another limitation of the current CDTI programmes is the exclusion of children under-five (< 90 cm or < 15 kg). It is critical to develop a paediatric formulation of anti-*O. volvulus* treatment that can be safely distributed to this age group in areas with high risk of OAE.

### Epilepsy treatment and management in onchocerciasis-endemic areas

Onchocerciasis-associated epilepsy does not manifest very differently from other forms of epilepsy and, as such, does not require distinct specialised services. However, epilepsy services are often unavailable in remote rural onchocerciasis-endemic areas where OAE is prevalent, or they function sub-optimally due to a shortage of trained and motivated healthcare providers, interrupted drug supplies, and/or lack of diagnostic equipment. Hence, there is a need to strengthen or initiate epilepsy diagnostic and treatment services in OAE-affected areas. We recommend that Ministries of Health in these areas decentralize epilepsy services and organize training for primary healthcare providers, including non-physicians, to diagnose and treat epilepsy.

Onchocerciasis elimination programmes can serve as an effective advocacy platform to scale up the treatment for people with epilepsy (PWE) in accordance with WHO guidelines, utilising primary healthcare workers as key service providers. Additionally, ivermectin community drug distributors (CDDs) could undergo training to identify individuals suspected of having epilepsy during house-to-house censuses and advise them to seek care at an epilepsy treatment centre. Good collaboration and communication between onchocerciasis elimination and brain health programmes are crucial (Box 1).

OAE can be treated with low-cost ASMs, such as phenobarbital and carbamazepine. However, it is essential to provide ASMs free of charge to PWE, because their cost can lead to frequent treatment interruptions, particularly in already impoverished families. Establishing an efficient supply line of ASMs is critical, but this proves challenging in onchocerciasis-endemic areas due to their remoteness, weak healthcare infrastructure, financial constraints, and common security problems. A vital step would be to integrate the supply of ASMs into the regular supply of other essential medicines (antibiotics, antimalarials, painkillers) to primary healthcare facilities.

Currently, multinational pharmaceutical companies manufacturing ASMs show little or no engagement with initiatives aimed at improving the treatment and care of PWE in sub-Saharan Africa, arguably because of limited evidence. Therefore, conducting clinical trials and robust cohort studies to assess the effectiveness of ASMs in PWE in Africa is needed. While the ASM levetiracetam was recently added to the WHO Model Lists of Essential Medicines, its role in the treatment of certain forms of OAE requires thorough investigation.

Box 1. Recommendations for the WHO Onchocerciasis related recommendations

- In onchocerciasis-endemic areas with high epilepsy prevalence, the coverage of CDTI should be critically reviewed and urgent actions taken to increase it, if found to be sub-optimal.
- Update the existing WHO documentation constantly to reflect the ever-growing evidence on OAE.
- Recognise the need to incorporate the validated OAE case definition into onchocerciasis disease burden estimation and into programmatic decisions to identify areas requiring additional support/interventions.
- Involve OAE-affected communities in planning and intensifying onchocerciasis elimination strategies.
- Propose the development of a paediatric formulation of anti-*O. volvulus *treatments that can be safely distributed to children below five years of age.
- Advocate for the training of community drug distributors to recognise suspected cases of epilepsy.

Brain Health related recommendations

- Conduct studies to determine the population attributable fraction of the different causes of epilepsy in onchocerciasis-endemic areas.
- Contribute to formalise the case definition of OAE for epidemiological and burden of disease studies.
- Increase awareness of OAE by providing a voice to those affected by this condition at neurology conferences and other relevant forums.
- Organize epilepsy healthcare training campaigns in regions where OAE is reported.
- Improve the efficiency of the supply chain for ASMs to reach onchocerciasis-endemic villages where OAE is prevalent.
- Take into consideration the epilepsy prevalence in different areas during the distribution of ASM such that the demand and supply of ASMs will be matched adequately.

### Importance of disseminating evidence-based information about OAE

Communities must receive evidence-based information about OAE. The main message is that OAE is a preventable condition if children take ivermectin at least once a year. However, it should be clear that ivermectin is not a cure for those who have epilepsy. Good CDTI therapeutic coverage will reduce the incidence of OAE. However, communities and health care workers in OAE affected areas should be aware it will take a long time before the prevalence of OAE will decrease. Therefore, long-term support and treatment for persons with OAE will be needed. The frequent clustering of epilepsy in certain homes is often attributed to “bad spirits”, and there are misconceptions about transmission through close contact. It is expected that raising awareness about OAE will increase ivermectin uptake while reducing stigma and discrimination associated with epilepsy.

## Conclusions

To reduce the OAE disease burden, it is imperative to foster close collaborations between onchocerciasis elimination and brain health programmes at community, national, and international levels. Increased advocacy is needed to provide uninterrupted access to ASMs for all PWE in onchocerciasis-endemic areas. The WHO aims at increasing epilepsy services coverage by 50% from the coverage observed in 2021 [9]. Such a target is not very appropriate for remote onchocerciasis-endemic areas where few PWE take ASM in an uninterrupted way. In analogy with the HIV targets of “95% detected, 95% treated, 95% viral suppression” [10] we propose to adopt the 90-80-70 target proposed by the International League Against Epilepsy: 90% of all people with epilepsy are aware that their diagnosis is a treatable brain disorder, 80% of people with epilepsy have access to appropriate, affordable and safe ASM, and 70% of people with epilepsy on treatment achieve adequate seizure control [11].

Including OAE in the estimation of onchocerciasis disease burden is essential, as epilepsy would contribute both to years lived with disability and years of life lost. An increase in the number of disability-adjusted life years attributed to onchocerciasis would have the potential not only to intensify international efforts to eliminate onchocerciasis but also to secure funding for a comprehensive Morbidity Management and Disability Prevention (MMDP) programme for onchocerciasis, including OAE.

Combatting the stigmatisation and discrimination faced by PWE requires raising awareness in communities and promoting respect for human rights. Onchocerciasis is an easily preventable cause of epilepsy. Therefore, onchocerciasis elimination could help prevent the suffering and premature death of many.

The recently established WHO Global Program for Elimination of Onchocerciasis provides an excellent platform to raise OAE awareness to ensure that those suffering from epilepsy in onchocerciasis-endemic communities receive the care and treatment they deserve. A collective call is made to governments, civil societies, donors, pharmaceutical companies, and WHO to intensify efforts, increase investments, and actively participate in identifying and combatting preventable causes of epilepsy. Such endeavours would contribute considerably to improving the quality of life and preventing premature deaths for current and future generations.

## Data Availability

Not applicable.

## Abbreviations

ASMs: Anti-seizure medications
CDDs: Community drug distributors
CDTI: Community-directed treatment with ivermectin
HIV: Human immunodeficiency virus
ILEA: International League against epilepsy
MMDP: Morbidity management and disability prevention
OAE: Onchocerciasis-associated epilepsy
PWE: People with epilepsy
WHO: World Health Organization
